# The Emergency Severity Index, version 4, for pediatric triage: a reliability study in Tabriz Children’s Hospital, Tabriz, Iran

**DOI:** 10.1186/1865-1380-6-36

**Published:** 2013-10-02

**Authors:** Amir Hossein Jafari-Rouhi, Sara Sardashti, Ali Taghizadieh, Hassan Soleimanpour, Mohammad Barzegar

**Affiliations:** 1 Pediatric Health Research Center, Tabriz Children’s Hospital, Tabriz University of Medical Sciences, Tabriz, Iran; 2 Department of Emergency Medicine, Tabriz University of Medical Sciences, Tabriz, Iran

**Keywords:** Pediatrics, Triage, Emergency severity index, Reliability

## Abstract

**Background:**

The Emergency Severity Index (ESI) has earned reliability and validity in adult populations but has not been adequately evaluated in pediatric patients. The aim of this study was to assess the reliability of the ESI version 4 and inter-rater reliability measures to evaluate the performance of nurses in the emergency ward.

**Methods:**

Raters were part of the same team of pediatric emergency medicine team, including pediatric emergency medicine (PEM) physicians and pediatric triage (PT) nurses. Reliability and agreement rates were measured using kappa statistics. The measurements were compared with the admission rates, readmissions to the PEM division, location of admission and death as outcomes.

**Results:**

Initially, PT nurses rated 20 case scenarios. Further in a prospective cohort study, 1104 children were assigned ESI scores by both nurses and physicians. The ratings of case scenarios showed a kappa value of 0.84. In actual patients, ratings showed high concordance with the physicians’ ratings with the kappa value of 0.82 being in a good agreement with the nurses’ ratings. The main area of discordance was detected in level 4 where 48 cases were triaged in higher levels and 25 were triaged in lower levels. The analysis showed the likelihood of admission clearly increased as the ESI score decreased (*p<0.0001*). There was a significant correlation between the admission status and triage level in both PT nurses’ and PEM physicians’ ratings (*Spearman coefficient=0.374, 0.407; p<0.0001*).

**Conclusion:**

ESI scores assigned to the pediatric patients are reliable in the hands of experienced PT nurses and PEM physicians. The very good agreement between PT nurses and PEM physicians, demonstrated in this study, is essential in cooperative work in crowded referral emergency departments and helpful in challenging triage cases.

## Background

The Emergency Severity Index (ESI) is a promising five-level triage system being widely studied in adult populations, where it is supposed to sort patients according to their medical acuity and resource allocation. The first version was only used for adults, while pediatric vital signs were included in newer versions, and ESI version 4 (v.4) is designed to include triage patients of any age group [1-4].

Triage in children seems to be more challenging compared to adults because of their different response to physiological and psychosocial stressors [1]. Children are more vulnerable to a range of injuries such as dehydration and viral infections. Furthermore, due to the need for special communication skills, assessing the level of urgency and acuity of children’s clinical symptoms is known to be challenging [4]. Several studies have evaluated the efficacy of nurses in triage ratings in emergency departments where the mentioned differences make pediatric triage more challenging [5-7]. A study has found strong agreement among nurses in the use of the third version of the ESI in pediatric patients [8]. It has also been shown that there is a high rate of agreement between physicians and nurses in the implementation of the ESI v.4 [9]. However, there are several areas in which nurses have difficulty in the triage of pediatric patients using ESI v. 4, including infant triage [4].

The aim of this study was to compare the agreement rates between pediatric emergency medicine (PEM) physicians and pediatric triage (PT) nurses using ESI v.4 in the emergency department (ED) of Tabriz Children’s Hospital. To evaluate the general knowledge of PT nurses and to negotiate individual weak points in triage we planned an educational session before evaluation of their performance on 20 standard case scenarios. In another phase of the study, focus was on the agreement rates between PT nurses and PEM physicians using ESI v.4 on a considerable sample population to shed light on practical shortcomings.

## Methods

### Study design

We executed a review session on ESI v.4 implementation methods for their effective employment as a vital stage during the patient admission procedure in our ED. In the first phase of our study, PT nurses filled out questionnaires on 20 standard case scenarios to assess the reliability for the written cases before rating actual patients. In the second phase, we conducted a prospective cohort study on 1104 children who presented to our pediatric ED from August through December 2011. The study was approved by the Ethics Board of Tabriz University of Medical Sciences.

### Study setting and population

The study was performed at the ED of Tabriz Children’s Hospital affiliated with Tabriz University of Medical Sciences with approximately 45,000 annual visits to the emergency department. Cases were selected from all patients received on 3 random days of every week between 2 p.m. and 10 p.m. using a computer-based randomization system.

The patients who could not be followed and the ones who left the ED before being visited by a PEM physician were excluded from the study.

Raters were members of an experienced team including PEM physicians and PT nurses who had been working together for at least a year in the triage section of the pediatric ED.

### Study protocol

The Fourth version of the ESI was used for patient triage by 12 trained PT nurses and 4 PEM physicians. PEM physician had been working in the pediatric ED for at least 5 years.

In the ED the triage nurse would perform the rating, guiding patients in levels 1 and 2 to the CPR (cardiopulmonary resuscitation) unit of the ED and the others to the fast-track clinic. Patients were then blindly re-rated by the investigator, a PEM physician. All cases were followed for 24 hours .Patients discharged from the ED were followed up by phone.

### Measures and data analysis

Demographic information including age and sex, and follow-up variables including admission status, readmission to the pediatric ED, location of the admission and death were documented. For the data analysis, we used SPSS version 16.0. Frequency and percentages were used for quantitative variables, while mean and standard deviation were used for qualitative data. To establish the existing correlations, chi-square and paired t-tests were implemented. The inter-rater reliability between PT nurses and PEM physicians was measured by the Pearson correlation coefficient and kappa statistics. The relationship between each ESI level and admission site was evaluated using the chi-square test and the relationship between the admission status and related triage level was assessed using Spearman’s correlation coefficient.

### ESI v.4 Algorithm

ESI level 1 patients require immediate life-saving interventions or present with any of the following: unresponsiveness, apnea, pulselessness, intubation or severe respiratory distress. ESI level 2 patients show severe pain/distress, new onset confusion, lethargy or disorientation or have a high-risk complaint. Patients not meeting level 1 or 2 criteria are triaged according to the number of resources needed before discharge. Patients who need two or more, one or no resources are triaged as level 3, 4 or 5, respectively. In the case of exceeding the vital sign criterion, patients will be up-triaged to level 2. Pediatric fever considerations are also included in ESI v.4.

## Results

In total, 1,458 children younger than 18 years old were initially given scores by PT nurses; 153 could not be followed because of code mismatching or missing data, and 301 left the ED before being visited by the physicians (participation rate = 75%).

The age of patients in this study ranged from 0.1 to 205 months old (3 days to 17 years), with the mean age of 31.4 ± 34.91 months and median age of 16 months. Of the 1104 patients, 635 (57.5%) were male. Admitted and discharged patients were not significantly different considering mean age and gender proportions (*p=0.519, p=0.158*).

The inter-rater reliability measured in the 20 case scenarios completed by the PT nurses was 0.84 overall (95% CI: 0.84 to 1) (Table 1).

**Table 1:** Inter-rater reliability

**Group**	***Kappa (CI)***	***P-value***
**PT nurses**	0.84 (0.84 - 1)	<0.001
**PT nurses and PEM physicians**	0.82 (0.67 - 0.84)	<0.001

For actual patients, PT nurses’ and PEM physicians’ ratings are compared in Table 1. In 964 cases (87.3%), PT nurses and PEM physicians assigned similar triage levels. In 83 cases (7.5%) PT nurses rated patients more critically with lower triage levels compared to the physicians. Conversely, in 57 cases (5.2%) PT nurses assessed patients less critically giving higher triage levels. As shown in Table 1, the main area of discordance was in level 4 where 48 cases were triaged in lower levels and 25 cases were triaged in higher levels by PT nurses. In general, the PT nurses’ ratings showed high concordance with the PEM physicians’ ratings, is shown in Table 1 [kappa (measure of agreement)] = 0.82*; Pearson’s r= 0.91, p<0.0001*).

About 10.1% of all the evaluated patients were admitted during 24 hours. Table 2 shows the distribution of PEM physicians’ ESI ratings compared to admission status. Chi-square test revealed that the number of admitted patients in levels 4 and 5 was significantly reduced in PEM physicians’ triage (*χ2 = 49.43, df = 8, p<0.0001)*. Table 3 shows the distribution of PT nurses ESI ratings considering admission status. Similarly, chi-square test revealed a significant reduction in number of admitted patients in levels 4 and 5 (*χ2 = 45.12, df = 8, p<0.0001*) (Table 4).

**Table 2:** PT nurses’ and PEM physicians’ ESI ratings in the emergency department of Tabriz Children’s Hospital, Tabriz, Iran

**Assigned score**	**PT Nurses**					
**PEM physicians**	**Level 1**	**Level 2**	**Level 3**	**Level 4**	**Level 5**	**Total**
**Level 1**	6	0	0	0	0	6
**Level 2**	0	300	6	15	1	322
**Level 3**	0	4	96	11	4	115
**Level 4**	0	13	7	254	21	295
**Level 5**	0	4	6	48	308	366
**Total**	6	321	115	328	334	1104

**Table 3:** Distribution of PEM physicians’ ESI ratings compared with admission status, Tabriz Children’s Hospital, Tabriz, Iran

**Rating of PEM physicians**	**Discharge (%)**	**PEM Division or ward admission (%)**	**ICU admission or death (%)**
**Level 1**	0 (0.0)	0 (0.0)	6 (100)
**Level 2**	222 (69.0)	96 (29.8)	4 (1.2)
**Level 3**	113 (98.2)	2 (1.8)	0 (0.0)
**Level 4**	290 (98.0)	6 (2.0)	0 (0.0)
**Level 5**	366 (100)	0 (0.0)	0 (0.0)

**Table 4:** Distribution of PT nurses’ ESI ratings compared with admission status, Tabriz Children’s Hospital, Tabriz, Iran

**Rating of PT nurses**	**Discharge (%)**	**PEM division or ward admission (%)**	**ICU admission or death (%)**
**Level 1**	0 (0.0)	0 (0.0)	6 (100)
**Level 2**	228 (71.0)	90 (28.1)	3 (0.9)
**Level 3**	111 (96.5)	3 (2.6)	1 (0.9)
**Level 4**	319 (97.3)	9 (2.7)	0 (0.0)
**Level 5**	332 (99.4)	2 (0.6)	0 (0.0)

There was a significant correlation between admission status (ICU admission, telemetry admission, ED stay or death) and the related triage level in both the scores assigned by the PT nurses (*p<0.0001, Spearman correlation coefficient = 0.374)* and PEM physicians (*p<0.0001, Spearman correlation coefficient = 0.407*). All patients assigned to level 1 by both PEM physicians and PT nurses were admitted to the intensive care unit (ICU); two of these patients died in the first 24 hours. For level 2 patients, three cases (0.93%) categorized by PT nurses and four cases (1.24%) categorized by PEM physicians, were admitted to the pediatric ICU, and the rest were hospitalized in other wards or discharged.

## Discussion

The aim of this study was to establish the reliability of the ESI v4 in our pediatric emergency department when used by an experienced team of PEM physicians and PT nurses. In the first phase, the performance of PT nurses was acceptable; in the second phase, the ESI v4 was found to be a reliable tool in the ED triage section.

Several other studies have also found moderate to high rates of inter-rater reliability using the ESI v.4 in pediatric patients [5,10-12]. In one of the most extensive evaluations of ESI reliability in pediatric patients, 40 case scenarios and 100 actual patients were evaluated in each of five research sites. Raters had been selected from both pediatric and non-pediatric nurses. Specialized nurses were 31% less likely to assign improper ESI levels. Similar to our study, the inter-rater reliability was better for case scenarios (kappa = 0.77 vs. 0.84) compared to actual cases (kappa = 0.57 vs. 0.82). Another study reports high agreement rates among nurses and PEM physicians closer to those in this study, but the evaluation was exclusively based on case scenarios. In our study, the PT nurses performed well on the case scenarios. For actual patients, two experienced PEM physicians double-triaged the ratings the PT nurses had assigned with high agreement rates (kappa = 0.82). The better agreement rates measured point to the fact that PT nurses in the present study have good experience in the field, and specialization improves triage success and hence ED management.

In our study, 1104 actual pediatric patients were double-triaged at the bedside. Assessment of inter-rater reliability on actual patients is more rigorous in comparison with case scenarios [5,13]. It has also been stated that real time ESI ratings in the specific environment of the ED should be considered in agreement rate measurements [11]; we believe that the large number of actual patients evaluated in the present study make it unique and conclusive.

Furthermore, in the study of Travers et al., the main areas of inconsistency were found to be in the triage of the most and least acute patients, children aged less than 1 year and those with medical chief complaints [5]. In our study the main discrepancies were related to the choice of allocated resources for less critically ill patients, and the largest number of mistriaged patients was seen in level 4. Similar to another study, we propose that this indicates the PT nurses’ capability to recognize of critically ill patients, which is crucial in emergency settings [12]. However, we suggest these discrepancies propose the need for PEM physicians to update their nurse coworkers the choice of resources.

On the other hand, observation of patients’ distribution shows fewer cases assigned to level 1 and level 5. Other studies have concluded that this distribution pattern could indicate that ESI should be revised to give a better picture of the patient case-mix [7].

Reliability assessments are crucial in keeping the coherency of the dynamic ED environment [11]. We suggest that interprofessional education, the main precursor of “interdisciplinary collaboration,” which leads to role awareness and mutual trust and respect in teams [14], could be the subject of further research to improve triage tools such as the ESI.

Trust, being an essential element in collaborative teams [15], was the conceptual term authors had in mind in order to use the outcomes of this study to raise functional efficacy. The inter-rater reliability showing high concordance between the PEM physicians and PT nurses in a single center, who have been working together for a significant amount of time, supposedly illuminates a raw estimation of this conceptual term.

The ESI is a simple triage tool but proved to be more reliable than previous three-level ones. The psychological aspects described in detail in different studies show that the use of this triage tool cannot be carried out in a computerized way when it comes to decision -making in critical situations such as the ones encountered in the pediatric emergency department.

### Limitations

The present study was performed in 4 months. However, we believe the high rates of referrals from other parts of the country may compensate for this.

Furthermore, to fulfill the ethical considerations, all patients rated level 1 or 2 by PT nurses were immediately transferred to the CPR unit, and visited and triaged there by the PEM physician.

The new concept briefly discussed in our study is more theoretical. We did not use any questionnaires to obtain a subjective view of the collaborative result, but we think the whole objective is to suggest a different viewpoint.

We should state that nurse and physician competency criteria were the same as the hospitals' and not reestablished for the present study.

## Conclusion

Considering this study and previous research works, we can conclude that the ESI has proved to be reliable in pediatric patients. The main point of consideration in these studies seems to be the role of education and specialization. However, various study reports on measured agreement rates highlight the need to investigate triage tools from new perspectives.

## Competing interests

The authors declare that they have no competing interests.

## Authors’ contributions

AHJR was the leading author to propose the study aim, supervise study course, and prepare drafted manuscript. SS was involved in data acquisition, preparation of drafted and final manuscript, and performing essential revisions. AT, HS, and MB were involved in development of study methodology, supervision and final review of the manuscript. All authors read and approved the final manuscript.
